# 

**Published:** 1872-01

**Authors:** 


### TABLE OF CONTENTS.

ORIGINAL COMMUNICATIONS:

How to Extract a Diseased and Lost Eye. By Julian J. Chisolm, M.D., Baltimore, Maryland, Professor of Eye and Ear Surgery in the University of Maryland, etc. .......... 551

My Experience with a Carbuncle (Anthrax.) By Wm. A. Greene, M.D., Americus, Georgia, Vice-President of the Georgia Medical Association .......... 558

Convergent Strabismus. By Dr. C. A. Simpson, Atlanta, Georgia... 563

Atlanta Academy of Medicine. Reported by Jas. B. Baird, M.D.... 569

(Hygiene of Gestation. By Jas. B. Baird, M.D., Chairman of the Section on Hygiene, being part of a Report to the Atlanta Academy of Medicine.——Hygiene of Gestation. By Wm. H. Cumming, M.D., being part of a Report to the Atlanta Academy of Medicine from the Section on Hygiene.)

Abstracts from German Journals. By Charles Raushenberg, M.D., Atlanta, Georgia .......... 585

(Pathology of the Movable or Wandering Kidney. By Dr. Rudolph H. Ferber, of Hamburg, Germany.——Contributions to the Knowledge of Pharyngeal Diphtheritis. By Dr. A. Classen, at Rostock, Germany.)

Puncture of the Colon in Tympanitis. (Selected.) .......... 598

EDITORIAL AND MISCELLANEOUS:

Prospects for the New Year .......... 599

Medical Education .......... 600

Bibliographical .......... 601

Monatsschrift Für Ohrenheilkunde .......... 602

Dr. C. A. Simpson .......... 605

Miscellaneous .......... 606

Complete Inversion of the Bladder, and the Protrusion of the Organ at the Urethra.——Liniment for Fissure of the Anus.——A Case of Electrotherapy.——Bromide of Calcium.——On the Mode of Administering Opium in Delirium Tremens.——Aspirations of Gas and Fluid in Strangulated Hernia.——Carbuncle Treated by Cut Cups.——Ergotine as a Hæmostatic.——Tænia in an Infant Five Days Old.——Placenta Prævia Treated by Digital Compression.

